# The involvement of community pediatricians in the treatment of developmental-behavioral difficulties as perceived by directors of child development centers

**DOI:** 10.1186/s13584-021-00492-8

**Published:** 2021-10-18

**Authors:** Rachel Nissanholtz-Gannot, Davidovitch Michael, Yael Ashkenazi, Zachi Grossman

**Affiliations:** 1 Department of Health Systems Management, School of Health Sciences, Ariel University, Ariel, Israel; 2 Smokler Center for Health Policy Research, Myers-JDC-Brookdale Institute, Jerusalem, Israel; 3 Child Development, Medical Division and Research Institute, Maccabi Healthcare Services, Tel Aviv, Israel; 4 Kahn-Sagol-Maccabi Research and Innovation Institute, Maccabi Healthcare Services, Tel Aviv, Israel; 5 Adelson School of Medicine, Ariel University, Ariel, Israel; 6 Maccabi Healthcare Services, Tel Aviv, Israel

**Keywords:** Developmental-behavioral difficulties, Pediatricians, Developmental pediatrics, Israel

## Abstract

**Background:**

Developmental-behavioral issues are among the most frequent and disabling conditions of children and adolescents seen in ambulatory settings. Guidelines of the Israeli Pediatric Association and the Israeli Society for Developmental Pediatrics specify the role of the primary-care pediatrician in screening and early identification of mild developmental behavioral conditions and define the criteria for referral to child development institutes. The aims of this study were to examine and describe how directors of these institutes perceive the role and involvement of community pediatricians in child development.

**Methods:**

Qualitative interviews of the directors of 22 child development institutes from the ministry of health and the four health plans.

**Results:**

According to the interviewees, there is little involvement of community pediatricians in detecting developmental delays, and it is mainly nurses and preschool teachers who detect such delays. They report that the key barriers that deter community pediatricians from greater involvement in child development diagnosis and treatment are lack of time, lack of compensation, and insufficient clinical knowledge. The interviewees would like to see community pediatricians conducting the primary medical evaluation, providing parental guidance, referring to therapists in mild cases, exercising discretion before referring children to child development institutes and providing relevant information to the institutes in the referral process. The mechanisms that they proposed for increasing the involvement of community pediatricians were expansion of pediatricians’ training, increased pediatricians’ use of teleconsultation with child development specialists and incentives for thorough performance of developmental assessments.

**Conclusions:**

Due to the importance of the Issue, we strongly recommend that policymakers require child development principles, evaluation, and providing appropriate parental guidance in the curriculum of the Israeli pediatric residency program. In addition, health plans should compensate pediatricians who need to conduct longer visits for children with developmental delays. The health plans should also develop teleconsultation channels for pediatricians with child development specialists to reduce unnecessary referrals to child development institutes.

## Introduction

There is growing epidemiological evidence that developmental-behavioral (DB) issues are among the most frequent and disabling conditions of children and adolescents seen in ambulatory settings [1]. In the US, autism and early identification of developmental behavioral problems (DBP) have increased: 20% have chronic health conditions; 20% have mental health diagnoses, which are now the costliest chronic conditions of children [2]. In Australia, autism, attention deficit hyperactivity disorder (ADHD) and sleep disturbances are the most frequent diagnoses noted for children consulting a pediatrician [3]. In Israel, a recent survey conducted among members in The Israel Pediatric Research in Office Setting (IPROS) network demonstrated that 29% of the children in their care were diagnosed with developmental-behavioral conditions (personal communication).

The US Society for Developmental and Behavioral Pediatrics (SDBP) states in its action plan that children with the most complex developmental and behavioral issues should have access to highly qualified DBP professionals, and that children with less complex issues be able to receive high-quality care in the generalist setting, when appropriate [4]. The Israel Society of Pediatric Neurology and Development (ISPND) has also issued guidelines specifying the role of the primary-care pediatrician in screening and early identification of mild DB conditions [5]. In this and other studies among US pediatricians, barriers of time, training, and reimbursement for early identification of DBP conditions were frequently noted [6–8]. Similarly, in Israel, a study performed among parents and pediatricians concluded that pediatricians’ training and practice do not reflect mental health needs in the community. Limited training is associated with limited interest and limited involvement, and training plays a role in overcoming the barriers of time, knowledge, competence, confidence, and professional identity [3].

In response to the lack of training, the current 3-year pediatric residency program in the US requires at least a month’s rotation in child-development centers for exposure to DBP [9]. In Israel, the pediatric residency program does not require this although it one of the longest residencies (4.5 years). The Goshen initiative for improving child healthcare focuses on the establishment of continuous medical education courses in DBP for practicing pediatricians working in community pediatric clinics and in Maternal Child Health Clinics (MCHC) [10].

Israel has a system with three spheres of diagnosis and treatment for children with developmental difficulties, headed by child development institutes, in which most of the diagnosis and some of the treatments are done. There are more than 100 units and hundreds of service providers in these facilities. The institutes carry a heavy workload and waiting times for appointments are long. The 2017 State Comptroller’s Report noted that the waiting times for diagnosis and treatment greatly exceed Ministry of Health stipulations (up to 3 months) and may be as much as a year or more.

Referral to child development institutes is not direct. Parents who fear that their children have a developmental problem turn to the pediatrician first to receive a referral to the institute. This is a point at which the pediatrician, who serves as a gatekeeper, can also take the opportunity to be involved in the treatment process and contribute to it.

The perspectives of DBP professionals on the performance of general pediatricians in Israel with regard to the identification and management of DBP conditions have not been explored. The aims of this study were to examine and describe how directors of child-development (CD) institutes in Israel perceive the role and involvement of community pediatricians in the area of CD Among other things, we examined their perception of the desirable involvement of community pediatricians in the area of CD and the extent to which children are needlessly referred to CD institutes. We also asked them, if pediatrician involvement in CD is desirable, what training and other resources are required for them to be able to do so?

## Methods

The study employed qualitative methods. It rested on 22 interviews conducted by means of a semi-structured in depth questionnaire. The interviewees were directors in the area of CD and pediatrics at Israel’s four health plans and at the Ministry of Health (7), of CD institutes at the health plans (12), and at the ministry (3). (see also Myers-JDC-Brookdale report [11]).

Sampling was directed to include institute directors from all the health plans and from the ministry, from the center of the country and the periphery in the north and in the south. The directors were approached by an email letter that explained the purpose of the study and requested an interview.

The questions focused on the following topics: The nature of the directors’ relationships with various CD services in the community; the nature of their relationships with community pediatricians; the extent to which, in their view, children who did not require treatment were referred to the institutes; the extent to which, in their view, community pediatricians could and should be involved in the diagnosis and treatment of CD problems; and whether in order to do so, the latter required training or other resources. The interviews were conducted by telephone from January to May 2019 and recorded with the consent of the interviewees.

Content analysis was performed to identify key themes. The analysis included a thorough reading of the texts of the recorded interviews, and the identification of main- and sub-categories. The first interviews were analyzed separately by three investigators on the research team and followed by discussion to arrive at consensus on the initial categorization. For purposes of the analysis we used Narralizer software for qualitative research [12]. The program is an auxiliary tool, it does not replace the researchers’ judgment in the construction of categories but facilitates data classification and sorting.

The study was approved by the ethics committee of the Myers-JDC-Brookdale Institute.

## Results

Several themes and sub-categories were identified in the interviews.

- **Involvement of community pediatricians in CD**
  1. The current involvement of pediatricians
    There was broad agreement among the interviewees that there was little involvement of community pediatricians in the area of CD and that—by and large—pediatricians did not constitute a significant or contributing factor in the detection of children with developmental delays or the identification and treatment of the problem. Moreover, the directors added, many pediatricians are not at all convinced that involvement in CD was part of their proper purview, and they are quick to transfer patients to another source of treatment without performing the basic activities expected of them by the interviewees.
  2. The desirable involvement of pediatricians
    The interviewees would like to see community pediatricians more involved in the treatment of children with CD difficulties, notably in the following ways:
    1. Pediatricians should conduct the **primary medical evaluation** and refer children with problems to relevant preliminary tests—such practice would save precious time upon the child’s arrival at the CD institute and, in some cases, rule out problems that are unrelated to developmental delays (e.g., celiac disease or anemia). As one interviewee put it:
      I would like pediatricians to be the primary party to relate to the problem, just as they are the primary party to relate to any other medical problem (interviewee no. 12).
      For example, in cases of speech delay, pediatricians should send the child for a hearing test: The pediatrician would discuss the results with the parents and in the case of impairment, the child would be referred to the proper resource for treatment or for surgery (for a cochlear implant) obviating the long wait for an appointment at a CD institute. The interviewees noted that pediatricians generally serve as an important figure of authority for parents, and parents on the whole are responsive to a pediatrician’s recommendations regarding necessary tests or treatment.
    2. Community pediatrics as a resource for concerned parents and the provision of guidance. In the case of mild BD problems when there is no need for a CD professional, a knowledgeable pediatrician can provide the warranted guidance rather than refer a child to the institute. One example cited by many of the interviewees is congenital muscular torticollis as sometimes exhibited by infants, which can be remedied with the help of simple exercises.
    3. Direct referrals to therapists in mild cases, for instance, a Speech-Language Pathologist (SLP)—As evident from the interviews, most directors do not expect pediatricians to take it upon themselves to refer patients directly to therapists. However, a few did say that they would like pediatricians to be armed with that option (e.g., to physio- or SLP) in mild, defined cases such as a brief delay in the onset of walking or unclear pronunciation.
    4. Exercising discretion before referring a child to a CD institute—The interviewees would like pediatricians to refrain from automatic referrals at the request of parents or the recommendation of a preschool teacher or nurse at a Maternal Child Clinic. The pediatricians should form their own impression of the child and exercise their discretion. If need be, they should explain to the parents that there is no cause for referral to a CD institute.
      On this question, there was also a dissenting opinion. A few interviewees contended that since pediatricians lack sufficient knowledge of CD, they could potentially cause harm and, preferably, should not perform screenings at all.
    5. Transfer of relevant information to the CD institutes—Written referrals constitute a work tool for institute physicians. They require detailed referrals to help staff arrive at a diagnosis. The valuable information includes: The child’s state of health, prior illnesses, prior hospitalizations if any, illnesses in the family, and CD-related aspects such as growth curve, head circumference, and prenatal (pregnancy) history. Information on the family and its background is also required. In many cases, community pediatricians have known a family for some time and are cognizant of its circumstances, residential milieu, and child-rearing practices
- Involvement of other parties in the identification of children with developmental delays

As understood from the interviews, in most cases pediatricians are not the first party to detect developmental delays in their patients. The active parties in this respect (apart from parents) are mainly nurses at Maternal-Child Clinics and preschool teachers. These parties refer the children to the pediatricians, who then refer the children to the CD institutes. The attitude of the interviewees to detection by preschool teachers was not uniform, some spoke of unnecessary referrals. Nonetheless, most commended the nurses and preschool teachers, asserting that they knew the children well, that they encountered them when healthy, and were able to assess their daily behavior and identify problematic cases. The Maternal-Child Clinics, for their part, devote structured time to the performance of developmental tests. Some interviewees, in fact, cited an opposite problem: Pediatrician disparagement of referrals by nurses and preschool teachers, which resulted in their missing signs that do require attention. However, as described below in the section on problems that aggravate the overload of CD institutes, some pediatricians believe that preschool teachers occasionally refer children who do not require treatment there, not due to mistaken detection but in order to relieve the burden on the preschool. In their opinion, the children will receive medical treatment in order to calm them down.

The interviewees voiced their sense that the system of CD institutes sometimes serves as a solution for other systems suffering from shortages and lack of workforce, causing the overload at the institutes. The **education system** was cited in this connection as it suffers from overcrowding and is understaffed. One interviewee described the process that brings children to the institutes who should not be there:

A preschool teacher desiring the assistance of a special education preschool teacher in order to obtain help and ease her work somewhat, gets hold of the parents and tells them, ‘Listen, [your child] must have occupational therapy, there is a serious problem.’ […] and [whereas] the parents pass on questionnaires to me that show the child to be perfectly normal […] and should I say … that I am not referring them for diagnosis, I receive calls from a stressed parent twice a week: ‘But the preschool teacher said, the preschool teacher said.’ So it is not only that we receive unnecessary referrals, but we also perform unnecessary diagnoses, knowingly (interviewee no. 13).

The situation is similar in pediatric mental health, which suffers from a serious shortage of professionals and for which the CD institutes constitute a relatively accessible alternative. In the opinion of the directors, some referrals to the institutes stem from lack of knowledge on the part of pediatricians; rather than to an institute neurologist, the children should have been referred a psychiatrist. That being said, it was evident from the interviews that in early childhood, the boundary between the two areas is not always clear.

The interviewees mentioned an additional approach of early detection and response to mild problems. They suggested that CD professionals be introduced into Maternal-Child Clinics to provide brief interventions and determine whether to refer a child to the institutes or to offer short-term treatment at the clinics.

(iii) Barriers to the involvement of community pediatricians in CD diagnosis and treatment

The interviewees cited several key barriers which they believe deter community pediatricians from greater involvement in CD diagnosis and treatment: Lack of time, lack of compensation, and insufficient clinical knowledge.

Lack of time—Of all the barriers cited, the interviewees perceived the lack of time as the most severe and they related to it from several aspects:

They believe that lack of time is a constant for community pediatricians. Appointments are extremely overbooked and, during visits, for which only a few minutes are allotted, pediatricians are unable to check whether a child’s development is normal. Moreover, they are aware that other patients are waiting outside their door, which, in the directors’ opinion, only adds to the pressure of the pediatricians’ work and rules out their devoting the time needed to check a child’s development.

Moreover, pediatricians have no assigned time for routine check-ups of healthy children in their care. One check-up program initiated by a health plan did not meet with success. According to the directors, Israeli culture dictates that parents bring their children to a pediatrician only when ill. Healthy children do not visit pediatricians on a routine basis; and during a pediatrician’s examination for a cold or pneumonia, for instance, there is no real time to check a child’s development. The directors expressed an interest in instituting a different type of visit to community pediatricians.

Compensation strategy for pediatricians—The lack of time relates also to the type of compensation pediatricians receive. Some community pediatricians (an estimated 40%) are self-employed and paid according to the number of visits made to them. Thus, if a visit takes too long, they see fewer children and their payment is smaller. The interviewees affirmed that CD examinations are lengthy and pediatricians should be compensated for them. A minority opinion contended that the mere fact of compensation would itself encourage community pediatricians to address the topic.

Lack of knowledge—Some interviewees mentioned the barrier of lack of CD knowledge on the part of community pediatricians. At the time of writing, the pediatric residency programs in Israel take place in hospitals, with no emphasis on community pediatrics. A block rotation in CD is not mandatory for pediatric residents either. Trainees wishing to acquire the necessary knowledge of CD must do so at their own initiative. The position of the interviewees was that pediatricians should receive at least minimal training in the topic and they require basic instruction at the level of primary clinics (e.g., training in Speech-Language Pathologies (SLP)). However, given the heavy workload of community pediatricians, the directors were not optimistic that such training would be easy to implement.

Additionally, not only the young generation of physicians lack sufficient knowledge of CD. The same is true of older pediatricians, as noted by one of the interviewees:

There is a problem in Israel at the level of skill and training for primary medicine. Therefore, I would address it throughout the physician’s careers […]: That is, for older physicians – via the health plan’s system of onsite training; for medical students – via the faculties and pediatrics residency where most of the work should be done. I would create a syllabus that definitely exposes them to these topics so that a generation of pediatricians rises for whom this language is not foreign (interviewee no. 18).

The result of this is that physicians encounter problems in the area of CD and do not have the tools to address them.

They sit at a clinic in a neighborhood where for a decade they see no […] child requiring intensive care [yet…] dozens and hundreds of children requiring CD [care] and not once in five years have they visited a CD institute. You can transcribe this in large writing with an exclamation mark and send it straight to the Scientific Council.

(iv) Possible ways to increase the involvement of community pediatricians
  Several ways to increase the involvement of community pediatricians in the treatment of developmental and emotional problems were proposed in the interviews. The main suggestions were:
  1. Change of pediatric training. The reference here is to integrate CD into the pediatrics residency to expose pediatricians to the contents and familiarize them more closely with the area. Another suggestion was that pediatric specialists receive simple, basic training focusing on the milestones of development to enable them to conduct informed screening and to winnow out the children referred to the institutes without cause. Another suggestion was that special training be provided to physicians interested in the area and that, subsequently, they be allowed to devote some of their time to working with children suffering from mild problems and referred to them by other physicians. These physicians would not replace CD physicians; rather, they would constitute an interim step between pediatricians and the institutes, somewhat easing the latter’s overload.
  2. **Use of online consultation** by CD specialists for community pediatricians. This would help the latter provide a response to DB problems, obviating the need for some referrals to the institutes. One interviewee said that her health plan did indeed offer this resource and physicians did avail themselves of it.
  3. **Incentives for** community pediatricians to perform **periodic health reviews** with the help of validated tools. Physicians would complete a screening questionnaire for children at specific junctures and receive compensation for the time spent.

## Discussion

The CD directors interviewed in this study would like to see community pediatricians more involved in the area of child development, especially while focusing on activities based on their knowledge as primary pediatricians and their longstanding acquaintance with the families. Their activities could be beneficial and save time both for the institutes and for the children and their families.

In their view, it is the role of community pediatricians to refer a child for relevant tests and, in mild cases, to guide parents on how to address a problem. Some directors also thought that, in certain cases, primary pediatricians could refer a child directly to specialists.

The interviewees attributed great importance to the pediatricians’ long-standing acquaintance with the children and their parents, their relationship with the family and the fact that they are a figure of authority trusted by the parents. That is the basis of the patient-centered medical home model, which has a potential to optimize the availability and quality behavioral health services [13]. Nonetheless, some interviewees noted that the pediatricians saw only ill children and thus might not detect developmental delays, even severe ones.

These problems were cited as critical—the heavy workload of pediatricians does not allow them to provide an appropriate response to DB problems; they are not compensated in a manner that would encourage them to invest the necessary time in a child with DB difficulties; they are not exposed to the area of CD during their residency, and many of them lack the requisite knowledge to judge the severity of the problems brought to them. These barriers were also found by Horowitz and others in a study in the U.S. [8]. Some directors believe that there are also pediatricians who have no desire to address the topic and therefore refrain from performing even the simple activities incumbent on all physicians, for instance, a basic physical examination.

Pediatricians themselves had criticism of their residency, namely that it omits exposure to the community. In studies conducted in Israel and in the U.S. these perceptions arose and pediatricians resent that they have inadequate training in DBP [8, 14]: Whereas residencies take place solely (or nearly solely) in hospitals, most physicians subsequently work in the community. They arrive without having been exposed to the common problems of children in the community, including developmental delays. For them to be more involved in these problems, a way must be found to expose them to the problems and equip them with knowledge of this area. In order to increase their involvement in this field, action must be taken to change the structure of specialization in pediatrics in Israel and increase exposure to the community.

These barriers are common in other countries and the literature shows that the longer the doctors’ training in DB the more s/he will treats and addresses DB issues [15] and when pediatricians believe that DBP are part of their role as clinicians they tend to address the problem and to use the help of other clinicians [14].

Therefore, policymakers need to require the introduction of the area of CD into the curriculum of the pediatric residency. Similarly, it is important to find a way to impart CD knowledge to physicians who have completed their residencies, to expose them to CD as well.

Interviewees mentioned the lack of time a doctor has with the child as a significant barrier in diagnosing developmental problems. In the US, pediatricians have time allotted for preventive care time that could be utilized to detect DB and other problems [16]. In Israel, on the other hand, pediatricians do not have time allotted for preventive care with the exception of Maternal-Child Clinics [17].

In general, children see a community pediatrician when they are ill and suffering from an acute problem that demands attention and care [17, 18]. One health plan did attempt to introduce “well-child” visits but the initiative encountered difficulties and never took root [19].

Note, too, that Israel has populations with large families who find it difficult to organize matters in such a way as to permit visits to a physician unless strictly necessary. Culture plays a role as well; it is less common in Israel than other countries to see a physician for a consultation or guidance if the child is healthy. Pediatricians consequently see children when they are ill, and all attention is focused on the acute illness rather than on psychological or other matters.

Health Plans (who are the employers of pediatricians in the community) should consider giving pediatricians the option of conducting longer visits in the case of children suspected of suffering from developmental delays and compensating them for the added time.

DPB physicians cited the solution of online calls as one that can assist in both providing information to pediatricians and in an appropriate diagnosis. The use of online calls has become common in Israel recently following the outbreak of the Covid-19, although using on-line calls was described as potential practice in public health emergencies [20]. In the US, for example, many health programs use telemedicine/ online calls [21].

### Limitations

In this study, we interviewed specific people and heard their positions so that there is no certainty that these are representative of all CD directors. There are approximately 150 institutions and units. We asked 23 physicians to be interviewed and 22 has agreed. One could not find the time to be interviewed. Nevertheless, the sample of physicians was not small (22) and it drew on different localities—both the center and the periphery, both hospitals and the community—and we believe that their position does reflect the prevailing climate of the area of CD.

## Conclusion and recommendations for health-policy stakeholders

It is recommended that the Israel Pediatric Association, who is in charge of developing the residency program for pediatric residents, and the Scientific Council of the Israel Medical Association who is in charge of overviewing the residency programs in Israel, require the introduction of child development into the curriculum of the pediatric residency program. In addition, health plans should compensate pediatricians who choose to conduct longer visits for children with developmental delays. They should also develop teleconsultation channels for pediatricians with child development specialists, to reduce unnecessary referrals to child development institutes.

Similarly, it is important to find a way to impart CD knowledge to physicians who have completed their residencies, to expose them to CD as well.

Another recommendation is to give therapists authority to refer children directly to CD clinics and to update the pediatrician. This step might save precious time. In addition when a physician writes a referral it must include physical examination by the pediatrician.

We also recommend that health plans will offer to compensate pediatricians who choose to conduct longer visits for children with suspicion of developmental delays.

The era we live in, with the Covid-19, provides opportunities for the use of technology. We suggest using telemedicine, online calls and online consultation for pediatricians in order to use the knowledge and experience of DBP physicians. We believe that consultation channels (e.g., online) with CD specialists be developed and strengthened at the health plans to obviate unnecessary referrals. It is a challenge of the policy makers to make it happen.

Finally, we believe it important to complete the picture of pediatrician involvement in the area of CD with a study interviewing the pediatricians themselves.

## Data Availability

The datasets during and/or analysed during the current study available from the corresponding author on reasonable request.
